# Periodic fever syndromes in Eastern and Central European countries: results of a pediatric multinational survey

**DOI:** 10.1186/1546-0096-8-29

**Published:** 2010-12-02

**Authors:** Nataša Toplak, Pavla Dolezalovà, Tamas Constantin, Anna Sedivà, Srdjan Pašić, Peter Čižnar, Beata Wolska-Kuśnierz, Miroslav Harjaček, Mariana Stefan, Nicolino Ruperto, Marco Gattorno, Tadej Avčin

**Affiliations:** 1 Department of Allergology, Rheumatology and Clinical Immunology, University Children's Hospital, University Medical Centre Ljubljana, Slovenia; 2 Rheumatology Unit, Department of Paediatrics and Adolescent Medicine, General University Hospital in Prague, Czech Republic; 3 Unit of Pediatrics Rheumatology, 2nd Department of Pediatrics, Semmelweis University Budapest, Hungary; 4 Department of Immunology, University Hospital Motol and 2nd Medical faculty, Prague, Czech Republic; 5 Mother and Child Health Institute, Medical School, University of Belgrade, Serbia; 6 1st Pediatric Department, Comenius University Medical School, Children's University Hospital, Bratislava, Slovakia; 7 Children's Memorial Health Institute, Warsaw, Poland; 8 Children's Hospital Srebrnjak, Zagreb, Croatia; 9 University Children Hospital M.S. Curie, Bucharest, Romania; 10 2nd Division of Pediatrics, "G. Gaslini" Scientific Institute, Genova, Italy

## Abstract

**Objective:**

To analyze the prevalence of diagnosed and suspected autoinflammatory diseases in Eastern and Central European (ECE) countries, with a particular interest on the diagnostic facilities in these countries.

**Methods:**

Two different strategies were used to collect data on patients with periodic fever syndromes from ECE countries- the Eurofever survey and collection of data with the structured questionnaire.

**Results:**

Data from 35 centers in 14 ECE countries were collected. All together there were 11 patients reported with genetically confirmed familial Mediterranean fever (FMF), 14 with mevalonate-kinase deficiency (MKD), 11 with tumor necrosis factor receptor associated periodic syndrome (TRAPS) and 4 with chronic infantile neurological cutaneous and articular syndrome (CINCA). Significantly higher numbers were reported for suspected cases which were not genetically tested. All together there were 49 suspected FMF patients reported, 24 MKD, 16 TRAPS, 7 CINCA and 2 suspected Muckle-Wells syndrome (MWS) patients.

**Conclusions:**

The number of genetically confirmed patients with periodic fever syndromes in ECE countries is very low. In order to identify more patients in the future, it is important to organize educational programs for increasing the knowledge on these diseases and to establish a network for genetic testing of periodic fever syndromes in ECE countries.

## Introduction

In the last decade the etiologic background of several monogenic autoinflammatory syndromes has been identified including FMF, MKD, TRAPS, CAPS - CINCA, FCAS and MWS, Blau syndrome and PAPA. Specific mutations of the genes encoding proteins that modulate inflammasome activity have been discovered for these syndromes. Periodic fever syndrome in which no mutation has been found so far is PFAPA syndrome [1,2]. The spectrum of autoinflammatory diseases is still expanding with continuous reports of novel genetic syndromes [3,4]. With better understanding and genetic testing the number of patients diagnosed with periodic fever syndromes is increasing but there are probably many unrecognized cases especially in countries where these diseases are rare.

To our knowledge, the frequency of periodic fever syndromes in Eastern and Central European (ECE) countries has not been reported so far. In this study we aimed to identify the prevalence of diagnosed and suspected periodic fever syndromes and other autoinflammatory diseases in ECE countries.

## 2. Methods

We performed a cross-sectional survey on periodic fever syndromes in ECE countries. Two different strategies were used to collect data on patients with periodic fever syndromes: i) the Eurofever survey- a secured web-based questionnaire administered to 54 pediatric rheumatology centers in ECE countries, members of PRINTO, ii) collection of data with the structured questionnaire distributed by e-mail to the pediatric immunologists included in the "J project" which is a physicians' education campaign promoted by ESID in order to sensitize pediatricians in ECE countries to the recognition of immune-mediated diseases. Questionnaire was also sent to other pediatricians with an interest in rheumatology and immunology, not included in PRINTO or ESID networks, mainly working in the tertiary care centers of pediatric rheumatology and immunology in ECE countries. Two rounds of e-mails with structured questionnaires were sent for both initiatives.

In total, the questionnaire was sent to 69 physicians from 16 ECE countries, including Albania, Bulgaria, Bosnia and Herzegovina, Croatia, Czech Republic, Estonia, Hungary, Latvia, Lithuania, Macedonia, Montenegro, Poland, Romania, Serbia, Slovakia and Slovenia. According to the 2007 Eurostat data for each included country, it is estimated that 123.6 million people live in these countries [5].

The structured questionnaire was mainly focused on monogenic inflammatory diseases. In particular we asked each center about the number of patients with a genetically confirmed diagnosis of monogenic autoinflammatory disease, where the genetic testing was performed and the possibility of genetic testing within the country. A suspected case was defined on the basis of the positivity of diagnostic criteria, whenever available (such as for FMF [6]) and/or on the intention to test for a given gene associated with autoinflammatory disease. We were also interested in the numbers of patients with suspected syndromes. In its third part the questionnaire included queries on the number of patients with other diseases grouped under the term autoinflammatory.

## Results

All together we received data from 37 physicians from 35 centers (54% combined response rate for both surveys) from 14 ECE countries including Albania, Bosnia and Herzegovina, Bulgaria, Croatia, Czech Republic, Hungary, Latvia, Lithuania, Macedonia, Poland, Romania, Serbia, Slovakia and Slovenia. The estimated resident population in these countries is 121.5 million and the number of children 0-19 years is approximately 27.0 million. This data was obtained from the latest available statistical report for each participating country.

Among the physicians who responded to the survey the distribution of specialists was as follows: 14 pediatric rheumatologists, 8 pediatric immunologists and 11 pediatricians with interest in this field.

### Genetically confirmed patients with monogenic periodic fevers

The overall numbers of patients reported with FMF, MKD, TRAPS and CINCA in ECE countries is shown in Table 1. Five adult patients were included- 3 with MKD and 2 with TRAPS. No country reported more than 2 genetically confirmed FMF patients. The majority of MKD patients were from the Czech Republic (8). Patients carrying *TNFRSF1A *mutations were reported from the Czech Republic (4), Hungary (3), Poland (2), Latvia (1) and Slovenia (1). CINCA patients were reported from Hungary (2), Czech Republic (1) and Slovakia (1).

**Table 1:** Genetically confirmed and suspected cases of periodic fever syndromes in ECE countries and estimated number of patients per number of children 0-19 years.

Periodic fever syndrome	Genetically confirmed cases	Suspected cases	Total	Estimated number per number of children 0-19 years*
FMF	11	49	60	1/465.500

MKD	14	24	38	1/771.400

TRAPS	11	16	27	1/1.080.000

CINCA	4	7	11	1/2.454.500

* Adult patients were excluded from calculation.

FMF- familial Mediterranean fever

MKD- mevalonate-kinase deficiency

TRAPS- tumor necrosis factor (TNF) receptor associated periodic syndrome

CINCA- chronic infantile neurological, cutaneous and articular syndrome

One genetically confirmed case of Blau syndrome was reported from Croatia. Genetic testing was done in Portland, Oregon, USA.

Genetic testing for suspected FMF patients from Slovenia was performed at the University Children's Hospital Ljubljana and for patients from Macedonia and Serbia at the Medical faculty Skopje. At the time of our survey, no genetic testing for other autoinflammatory diseases was available in ECE countries. Six centers from ECE countries sent DNA for genetic testing for periodic fever syndromes to Genova (Italy), two centers to Germany, including one to University of Freiburg, two centers to Turkey, one center to Montpellier (France), London (UK), Roma (Italy) and Barcelona (Spain).

### Suspected cases of monogenic periodic fever syndromes and reported cases of other autoinflammatory diseases

The majority of patients with suspected FMF were from Romania (13), followed by Poland (8), Bulgaria (5), Hungary (4) and Croatia (4). Two adult patients were included. Other countries reported 3 or less patients with suspected FMF.

Among suspected MKD patients the highest number was reported from Romania (5), followed by Serbia (4), Czech Republic (3) and Slovakia (3).

Among suspected TRAPS patients the highest number was reported from Poland (6) followed by the Czech Republic (3). One suspected TRAPS patient was reported from Albania, Croatia, Hungary, Latvia, Romania, Serbia and Slovakia.

On the basis of their clinical presentation some patients were included in the study as suspected CAPS. Three suspected CINCA cases were referred from Poland and one from the Czech Republic, Hungary, Latvia and Slovakia. Two suspected FCAS patients were reported from Croatia and one from Serbia. One suspected MWS case was reported from Latvia and Poland. One suspected PAPA case was reported from the Czech Republic and one suspected case with Blau syndrome from Hungary.

The data about other reported autoinflammatory diseases are presented in Table 2. Two adult patients were included- 1 PFAPA patient and patient with Schnitzler syndrome.

**Table 2:** Other reported autoinflammatory diseases

Autoinflammatory disease	Reported cases
PFAPA	282

Behçet disease	26

CRMO	23

Idiopathic recurrent pericarditis	2

Schnitzler syndrome	1

PFAPA- periodic fever syndrome with aphthous stomatitis, pharyngitis and cervical adenopathy

CRMO- chronic recurrent multifocal osteomyelitis

## Discussion

Published studies investigating periodic fever syndromes were mainly conducted in Mediterranean countries, western European countries and United States. To our knowledge, there has been no systematic evaluation of patients with periodic fevers in ECE countries.

The aim of this study has been to find out if periodic fever syndromes and other autoinflammatory diseases are recognized in ECE countries. We contacted all physicians from eastern and central Europe who are members of PRINTO and ESID (J-project for eastern and central European countries) and pediatricians with an interest in rheumatology and immunology working in the tertiary care centers of pediatric rheumatology and immunology in ECE countries. The response rate for surveys was 54% which is a possible limitation to the collected data. We believe that the main reason for this is linked to the low awareness of these diseases in ECE countries.

The estimated frequency of FMF patient in ECE countries appears exceedingly low comparing to the data from Western European countries and it is likely that many FMF patients remain unrecognized. It is possible that clinical presentation of FMF is milder or different in ECE countries because of environmental modifiers. The estimated frequency of FMF in Mediterranean population ranges from 1/256 among North African Jews to 1/500 among Armenians and Israelis [7]. Lower but significant frequencies have been reported in other countries around the Mediterranean Sea [8,9]. In some patients with clinical features of FMF no mutation of the *MEFV *gene could be identified. In the Western European countries, only a minority of patients' compatible with FMF present *MEFV *mutations [8].

Genetically confirmed MKD was in our survey reported in 14 patients, which is the highest number among all genetically confirmed periodic fever syndromes in ECE countries. Moreover, an MKD was suspected in further 24 cases. According to these data, it seems reasonable that MKD may play a relevant role as a possible cause of inherited periodic fever syndromes in ECE countries, as already observed in populations other than Dutch or north-European [10-12].

The number of patients with recurrent fever carrying one mutation of *TNFRSF1A *gene in ECE countries was equal to that observed for genetically confirmed FMF cases. TRAPS was originally considered an exceedingly rare disease with only few families described in the literature. During the past decades more than 50 disease associated mutations have been identified in hundreds of patients with different ethnic backgrounds [13]. Since the survey was not aimed to collect information on the type of mutations found for each patient we cannot exclude that part of the mutations found were hypomorphic variants of the gene, such as R92Q or P46L.

CINCA is a very rare congenital inflammatory disease [14] and we were able to identify only four patients from ECE countries.

In the present study, a relevant number of patients were classified as PFAPA. It is likely that at least part of the 282 patients with a PFAPA phenotype identified in ECE countries might also be positive at genetic testing [15].

In the majority of patients included in the survey genetic analysis was not done. With the effort of the Eurofever project http://www.printo.it/eurofever the network of laboratories that offer genetic testing for periodic fever syndromes was established. The next important step is to further spread the knowledge about these rare diseases among physicians of different specialties in ECE countries. A low number of trained pediatric rheumatologists and immunologists in these countries is likely one of the contributing factors for low recognition of these diseases. With the purpose to improve the knowledge about autoinflammatory diseases in the region of ECE countries and southern Europe, the first educational meeting was organized in October 2009 in Ljubljana, Slovenia. The meeting was officially supported by the PReS and the J Project, and was attended by 121 participants from 22 countries.

In summary, this study presents the first data on the prevalence of diagnosed and suspected autoinflammatory diseases in ECE countries. It demonstrates that all recognized periodic fever syndromes do affect also patients from ECE countries, with a frequency which appears to be lower than in the Mediterranean and Western European countries. In order to identify more patients in the future, it is important to continue with educational activities, that is one of the main aims of the Eurofever project.

## Abbreviations

FMF: familial Mediterranean fever; MKD: mevalonate-kinase deficiency (MKD); TRAPS: tumor necrosis factor receptor associated periodic syndrome; CAPS: diseases from the spectrum of cryopyrin associated periodic fever syndromes; CINCA: chronic infantile neurological cutaneous and articular syndrome; FACS: familial cold autoinflammatory syndrome; MWS: Muckle-Wells syndrome; PAPA: pyogenic sterile arthritis, pyoderma gangrenosum and acne syndrome; PFAPA: periodic fever syndrome with aphthous stomatitis, pharyngitis and cervical adenopathy; CRMO: chronic recurrent multifocal osteomyelitis; PRINTO: Paediatric Rheumatology International Trials Organization; ESID: European Society for Primary Immunodeficiencies

## Competing interests

The authors declare that they have no competing interests.

## Authors' contributions

NT, MG and TA participated in the design of the study. All authors participated in the acquisition of data from their countries. NT, NR, MG and TA participated in the statistical analysis and interpretation of data. NT, PD, MG and TA drafted the manuscript. All authors read and approved the final manuscript.
